# Fronto-Limbic Brain Dysfunction during the Regulation of Emotion in Schizophrenia

**DOI:** 10.1371/journal.pone.0149297

**Published:** 2016-03-01

**Authors:** Shaun M. Eack, Jessica A. Wojtalik, Scott M. Barb, Christina E. Newhill, Matcheri S. Keshavan, Mary L. Phillips

**Affiliations:** 1 School of Social Work, University of Pittsburgh, Pittsburgh, Pennsylvania, United States of America; 2 Western Psychiatric Institute and Clinic, University of Pittsburgh School of Medicine, Pittsburgh, Pennsylvania, United States of America; 3 Massachusetts Mental Health Center Public Psychiatry Division of the Beth Israel Deaconess Medical Center, Harvard Medical School, Boston, Massachusetts, United States of America; University of Tuebingen Medical School, GERMANY

## Abstract

Schizophrenia is characterized by significant and widespread impairments in the regulation of emotion. Evidence is only recently emerging regarding the neural basis of these emotion regulation impairments, and few studies have focused on the regulation of emotion during effortful cognitive processing. To examine the neural correlates of deficits in effortful emotion regulation, schizophrenia outpatients (*N* = 20) and age- and gender-matched healthy volunteers (*N* = 20) completed an emotional faces n-back task to assess the voluntary attentional control subprocess of emotion regulation during functional magnetic resonance imaging. Behavioral measures of emotional intelligence and emotion perception were administered to examine brain-behavior relationships with emotion processing outcomes. Results indicated that patients with schizophrenia demonstrated significantly greater activation in the bilateral striatum, ventromedial prefrontal, and right orbitofrontal cortices during the effortful regulation of positive emotional stimuli, and reduced activity in these same regions when regulating negative emotional information. The opposite pattern of results was observed in healthy individuals. Greater fronto-striatal response to positive emotional distractors was significantly associated with deficits in facial emotion recognition. These findings indicate that abnormalities in striatal and prefrontal cortical systems may be related to deficits in the effortful emotion regulatory process of attentional control in schizophrenia, and may significantly contribute to emotion processing deficits in the disorder.

## Introduction

Schizophrenia is characterized by significant impairments in the processing and regulation of emotion [1]. Stress and emotion dysregulation are both prominent contributors to outcome in people with the disorder [2, 3], and while their experience of emotion is similar to others [4], growing evidence is accumulating that individuals with schizophrenia have considerable difficulty in regulating their emotional experiences [5]. Challenges in emotion regulation have important implications for functional outcomes, given the contribution of managing emotions to role performance [6] and successful interpersonal interactions [7].

Current models of emotion regulation have outlined two broad domains of investigation: (1) an effortful (i.e., voluntary) domain where the regulation of emotion is conscious and explicit, and (2) an automatic domain where little to no conscious effort is involved in modulating emotional responses [8, 9, 10]. Phillips and colleagues outlined an influential neural model of emotion regulation which posited three effortful subprocesses consisting of suppression, reappraisal, and voluntary attentional control, each with their own supporting neural mechanisms [9]. Voluntary attentional control may be a particularly important subprocess of emotion regulation to understand in schizophrenia, given evidence of cognitive and attentional impairment in the disorder [11]. This subprocess was defined by Phillips et al. as consisting of "(1) selective attention, to direct or redirect attention toward goal-related stimuli, and (2) inhibition, to distract from goal-irrelevant stimuli" [9]. Other influential models of emotion regulation have identified similar effortful subprocesses, including "attentional deployment" outlined by Gross [8], and assessments of this subprocess often involve completing cognitive tasks while attempting to inhibit attention to task-irrelevant emotional stimuli [9, 12]. Neural networks shown to be involved in the voluntary attentional control subprocess of emotion regulation include the dorsolateral prefrontal, anterior cingulate, and orbitofrontal cortices, amygdala, paralimbic regions, and the parietal cortex [9].

Unfortunately, the neural basis of voluntary attentional control deficits related to effortful emotion regulation in schizophrenia have remained largely unknown, making precise treatment targets difficult to discern for intervention developers seeking to address problems in this domain. A small number of studies have begun to investigate the neural correlates of voluntary emotion regulation deficits, generally, in this population. Becerril and Barch [13] used an n-back paradigm with emotional faces as target stimuli and found dorsolateral prefrontal, hippocampal, and basal ganglia hyperactivity in response to faces of negative emotions in patients with schizophrenia. Anticevic and colleagues conducted a series of studies using negative emotional distractors during working memory tasks, and found that patients with schizophrenia fail to demonstrate appropriate prefrontal recruitment in response to negative distractors, perhaps due to altered fronto-limbic connectivity [14, 15, 16]. Another study by van der Meer and colleagues [17] found that patients with schizophrenia demonstrated significantly reduced ventromedial prefrontal and medial-temporal brain activity when attempting to reappraise negative emotion-provoking photographs. These findings suggest the feasibility of identifying neural signatures associated with deficits in the effortful regulation of emotion in schizophrenia, and indicate that negative emotional stimuli may produce significant fronto-limbic interference to the voluntary attentional control subprocess of emotion regulation. However, findings have been mixed, the impact of positive emotions has yet to be fully examined, and no information is available on whether disruptions in voluntary attentional control are associated with behavioral disturbances in emotion processing frequently observed in the literature [18].

This study employed an emotional faces n-back task widely used in affective disorders [19, 20] to examine neural abnormalities in the voluntary attentional control subprocess of the effortful emotion regulation processing stream of patients with schizophrenia relative to healthy volunteers. We hypothesized that relative to healthy controls, individuals with schizophrenia would demonstrate significant fronto-limbic hyperactivity in response to emotional face distractors when completing a working memory task, and that this hyperactivity would be related to behavioral deficits in emotion processing.

## Materials and Methods

### Participants

Study participants included 20 outpatients with schizophrenia or schizoaffective disorder and 20 age- and gender-matched healthy volunteers. Patients with schizophrenia who were 18 to 50 years of age, who were diagnosed with schizophrenia or schizoaffective disorder verified by the Structured Clinical Interview for DSM-IV (SCID) [21], who were psychiatrically stable and adherent to prescribed antipsychotic medications, and who had a IQ > 80 were recruited for a study of social-cognitive brain function in the disorder. Healthy volunteers were included if they were free from a current psychiatric disorder according to the SCID. MRI contraindications, a history of medical disorders producing cognitive impairment, significant neurological conditions, persistent suicidal/homicidal behavior, or significant substance abuse problems were exclusion criteria for both patients with schizophrenia and healthy controls. Table 1 presents demographic and clinical characteristics of the study sample. Patients were younger, the majority (55%) were in the first 8 years of the condition, and none were experiencing their first episode of psychosis. Patients and controls were well-matched with regard to age and gender, but healthy individuals were significantly more likely to be white and had higher IQ scores than those with schizophrenia.

**Table 1:** Demographic and Clinical Characteristics of Individuals with Schizophrenia and Healthy Volunteers.

	Healthy Volunteer (*N* = 20)	Schizophrenia (*N* = 20)	
Variable	*M* (SD)	*M* (SD)	*p*^a^
Age	26.50 (5.82)	27.80 (6.61)	.513
Male, *N* (%)	13 (65%)	14 (70%)	1.000
White, *N* (%)	16 (80%)	7 (35%)	.010
Attended College, *N* (%)	18 (90%)	15 (75%)	.407
IQ	106.55 (6.67)	97.90 (8.11)	.001
Diagnosis, *N* (%)			-
Schizophrenia	-	9 (45%)	
Schizoaffective Disorder	-	11 (55%)	
Illness Duration, yrs	-	4.85 (3.18)	-
Receiving second generation antipsychotic, *N* (%)	-	18 (90%)	-
Antipsychotic dose, cpz equivalence	-	308.08 (235.89)	-
BPRS [36] Total	21.90 (1.59)	38.05 (10.42)	< .001
Global Assessment Scale [37]	86.35 (6.67)	54.30 (11.85)	< .001

*Note*. BPRS = Brief Psychiatric Rating Scale

^a^Results of independent sample *t*-tests or Fisher's exact test, two-tailed

### Emotional Faces N-Back Task

Brain functions supporting the voluntary attentional control subprocess of effortful emotion regulation were assessed using an emotional face n-back paradigm [20]. An n-back task is a working memory task that asks participants to respond when they view a stimulus that is identical to a stimulus presented *n* trials previously. A 0-back task is primarily an target detection task, where participants are asked to identify a target stimulus as it is presented (e.g., respond when you see the letter M). A 2-back task is an effortful working memory task, where participants are asked to identify whether the current stimulus is identical to that presented two trials previously (e.g., A—B—A). This emotional faces n-back task consists of a standard n-back paradigm with 0- and 2-back conditions presented in random order. To introduce emotional information into the task, to which attention must be voluntarily controlled, n-back blocks randomly include happy, fearful, neutral, or no face distractors flanked on each side of target n-back stimuli (letters). Participants are instructed to ignore the emotional distractors and simply to focus on completing the cognitive demands of the working memory task. In this way, participants are required to engage in effortful emotion regulation to direct their attention away from irrelevant emotional stimuli to complete the n-back task. Emotional face stimuli consisted of grayscale happy, fearful, or neutral faces from the NimStim dataset [22], normalized for size and luminance, and balanced by gender. Each task block consisted of 12 trials with n-back and emotional distractor conditions randomly distributed across blocks. All blocks began with n-back instructions for the block presented for 3500ms, followed by individual trials of the n-back stimuli (with happy, fearful, neutral, or no emotional distractors) for 500ms and an interstimulus interval jittered at an average of 3500ms. All emotional distractor conditions were presented once for 0- and 2-back conditions for an approximate total task time of 6 minutes and 56 seconds. The 0-back condition is included primarily to reduce response habituation during 2-back trials, and since the focus of this research is on examining the impact of emotional distractors on effortful non-emotional cognitive performance, analyses were limited to the 2-back condition.

### Behavioral Measures of Emotion Processing

To examine the association between neural response to the effortful regulation of emotion and behavioral measures of emotion processing, emotion perception and emotional intelligence data were collected using the Penn Emotion Recognition Test [23] and the Mayer-Salovey-Caruso Emotional Intelligence Test (MSCEIT) [24]. The Penn Emotion Recognition Test is a 40-item forced-choice measure of facial emotion perception, where participants are asked to match an emotional label with happy, sad, fearful, angry, or neutral faces. The MSCEIT is a 141-item performance-based measure of the four domains of emotional intelligence outlined by Salovey and Mayer [25]. The instrument is performance-based in that participants are asked to solve emotional problems, which are scored based on normative responses, rather than to self-report on their emotional abilities. Previous studies have found both the Penn Emotion Recognition Test and the MSCEIT to have acceptable psychometric properties in healthy and psychiatric samples [26, 27, 28].

### Image Acquisition and Processing

Neuroimaging data were collected on a 3-T Siemens Tim Trio scanner with 12-channel head coil. Functional MR data were acquired using an echo T2*-weighted sequence with real-time motion correction (voxel size of 3.2 x 3.2 x 3.1mm, TR = 2000ms, TE = 28ms, bandwidth = 3004 Hz/px, flip angle = 90˚, FOV = 205mm, 64 x 64 matrix, 39 slices, slice thickness = 3.1mm). Structural MR data used for normalization were collected using a 3D MPRAGE sequence in the axial orientation (voxel size of 1.0mm, TR = 2200ms, TE = 3.31ms, flip angle = 9˚, FOV = 256mm, 256x192 matrix, 192 slices, slice thickness = 1.0mm). Functional images were inspected for significant motion or other artifacts, preprocessed in Statistical Parametric Mapping software, version 8 (Wellcome Department of Cognitive Neurology, Institute of Neurology, London, UK), and smoothed using an 8mm Gaussian kernel. A standard indirect normalization pipeline was used to normalize functional MR data based on each participant's own structural data, with the exception of two participants (1 patient and 1 control) whose structural data were inadequate for normalization and for whom preprocessing relied upon a standardized echo-planar image template provided by SPM.

### Procedures

Participants were recruited from Western Psychiatric Institute and Clinic and surrounding community clinics in Pittsburgh, PA. Upon recruitment, potential participants were screened for eligibility using the SCID and Ammon's Quick Test [29]. Participants then completed MRI procedures, including the emotional n-back task, and on a separate day behavioral measures of emotion processing by trained research technicians. All participants were trained on the completion of the different n-back conditions outside of the scanner on a practice computer before neuroimaging data collection. This study was approved and reviewed annually by the University of Pittsburgh Institutional Review Board, and all individuals provided written, informed consent prior to participation. Decisional capacity to provide informed consent was evaluated by trained research staff, and any participants incapable of understanding and communicating the research procedures, risks, and benefits to these staff were excluded from the study. Parental or guardian consent was not pursued in such cases, and given the medication adherence and psychiatric stability inclusion criteria for the study, no potential participants needed to be excluded due to inability to provide informed consent.

### Data Analysis

Differences in fronto-limbic brain function during the emotional faces n-back task between patients with schizophrenia and healthy controls were examined using general linear models constructed in SPM 8. After preprocessing, first-level task models were then constructed using condition onset times as primary predictors and BOLD signal response as the dependent variable, with motion parameters and BOLD signal outliers included as confounding covariates using the Artifact Detection Tools [30]. All trials, regardless of accuracy, were included in first-level models. Subsequently, second-level region of interest models were constructed to examine participant group (schizophrenia vs. control) x condition (happy, fearful, neutral) pairwise interactions, which included all study participants, regardless of task performance. First-level contrasts used to represent these conditions compared the specified emotional face trials to no face trials (e.g., happy—no faces, fearful—no faces) within the same n-back level (0- or 2-back). Fronto-limbic regions of interest were specified using the Wake Forest University PickAtlas toolbox [31], with regional definitions outlined by Tzourio-Mazoyer and colleagues [32] and included the bilateral amygdala, striatum (caudate, putamen, and pallidum), orbitofrontal, dorsolateral prefrontal, ventromedial prefrontal, insular, and anterior cingulate cortices, as well as the nucleus accumbens. Type I error was maintained at *p* = .05 using a combined voxel and uncorrected *p*-value threshold of *k* = 79 and *p* = .005, respectively, based on 10,000 Monte Carlo simulations using 3dClustSim based on AlphaSim [33]. All second-level fMRI models adjusted for between-group differences in age, gender (0 = female, 1 = male), IQ, and race (0 = non-white, 1 = white). Behavioral data were examined using generalized linear mixed-effects models, also adjusting for demographic and IQ confounders. Behavioral correlates of BOLD signal activity were investigated using within-group correlation models of the association between emotion processing measures and regional brain activity.

## Results

### Task Performance

Analysis of differences between individuals with schizophrenia and healthy volunteers on accuracy during the emotional n-back task indicated no significant differences between participant groups and no significant group by condition interactions, all *p* > .271 (see Table 2). Overall accuracy on the task was high, but as expected, significantly reduced in the 2-back compared to 0-back condition, χ^2^(1, *N* = 40) = 33.59, *p* < .001. Reaction time was also significantly slower for the 2-back versus 0-back condition, *F*(1, 1308) = 219.47, *p* < .001, with patients with schizophrenia demonstrating significantly greater response latency during 2-back trials compared to healthy individuals, *F*(1, 1308) = 13.05, *p* < .001. Two patients did not have reaction time data, as while they responded to the task, their target responses were incorrect. No other significant group, condition, or group by condition interactions were observed, and there were no significant differences in the accuracy or speed with which participants completed the task when emotional distractors were present.

**Table 2:** Emotional Faces N-Back Task Performance Among Individuals with Schizophrenia and Healthy Volunteers.

	Group			
	Healthy Volunteer (*N* = 20)		Schizophrenia (*N* = 20)	
Outcome	*M*	*SE*	*M*	*SE*
Accuracy (%)				
0-Back				
No Faces	97.80	1.43	95.91	1.89
Fearful Faces	98.39	1.15	96.67	1.57
Neutral Faces	98.37	1.16	96.34	1.71
Happy Faces	97.78	1.44	96.66	1.58
2-Back				
No Faces	94.89	2.72	92.69	3.15
Fearful Faces	91.99	3.87	92.80	3.11
Neutral Faces	94.94	2.70	93.08	3.00
Happy Faces	96.21	2.15	93.81	2.72
Latency (ms, log)^a^				
0-Back				
No Faces	6.27	.08	6.33	.08
Fearful Faces	6.32	.08	6.33	.08
Neutral Faces	6.32	.08	6.33	.08
Happy Faces	6.35	.08	6.34	.08
2-Back				
No Faces	6.45	.08	6.59	.08
Fearful Faces	6.47	.08	6.54	.08
Neutral Faces	6.52	.08	6.58	.08
Happy Faces	6.44	.08	6.62	.08

^a^Two participants were excluded from latency analyses, as while they responded to the task, their target responses were incorrect

### Fronto-Limbic Brain Function during Voluntary Attentional Control

Comparison of fronto-limbic BOLD signal response during the emotional faces n-back task for individuals with schizophrenia versus healthy controls indicated significant differential patterns of brain activity when completing 2-back trials during different emotional conditions. As can be seen in Table 3, patients with schizophrenia had significantly reduced right striatal activity in response to fearful versus neutral faces compared to healthy individuals. Further, those with schizophrenia exhibited greater right orbitofrontal cortex activity when presented with happy versus neutral faces compared to controls.

**Table 3:** Fronto-Limbic Differences in Brain Activity Between Individuals with Schizophrenia and Healthy Volunteers During the Emotional Faces N-Back Task.

	MNI Coordinates						
Contrast	(x, y, z)	Cluster Size	Location	BA	*t*	*p*	Direction
Fear—Neutral	14, 12, 6	113	Striatum	-	3.95	< .001	SZ < HC
Happy—Neutral	32, 24, -12	99	Orbitofrontal cortex	47	3.38	.001	SZ > HC
Fear—Happy	-14, 2, -6	255	Striatum	-	4.53	< .001	SZ < HC
	-34, 40, 2	178	Ventromedial prefrontal cortex, orbitofrontal cortex	47	3.94	< .001	SZ < HC
	26, 20, -2	131	Striatum	-	3.37	.001	SZ < HC
	40, 26, 12	113	Ventromedial prefrontal cortex	13	3.53	< .001	SZ < HC
	30, 30, -12	94	Orbitofrontal cortex	47	3.51	< .001	SZ < HC

*Note*. BA = Brodmann Area, HC = Healthy Control, SZ = Schizophrenia

**Fig 1:**
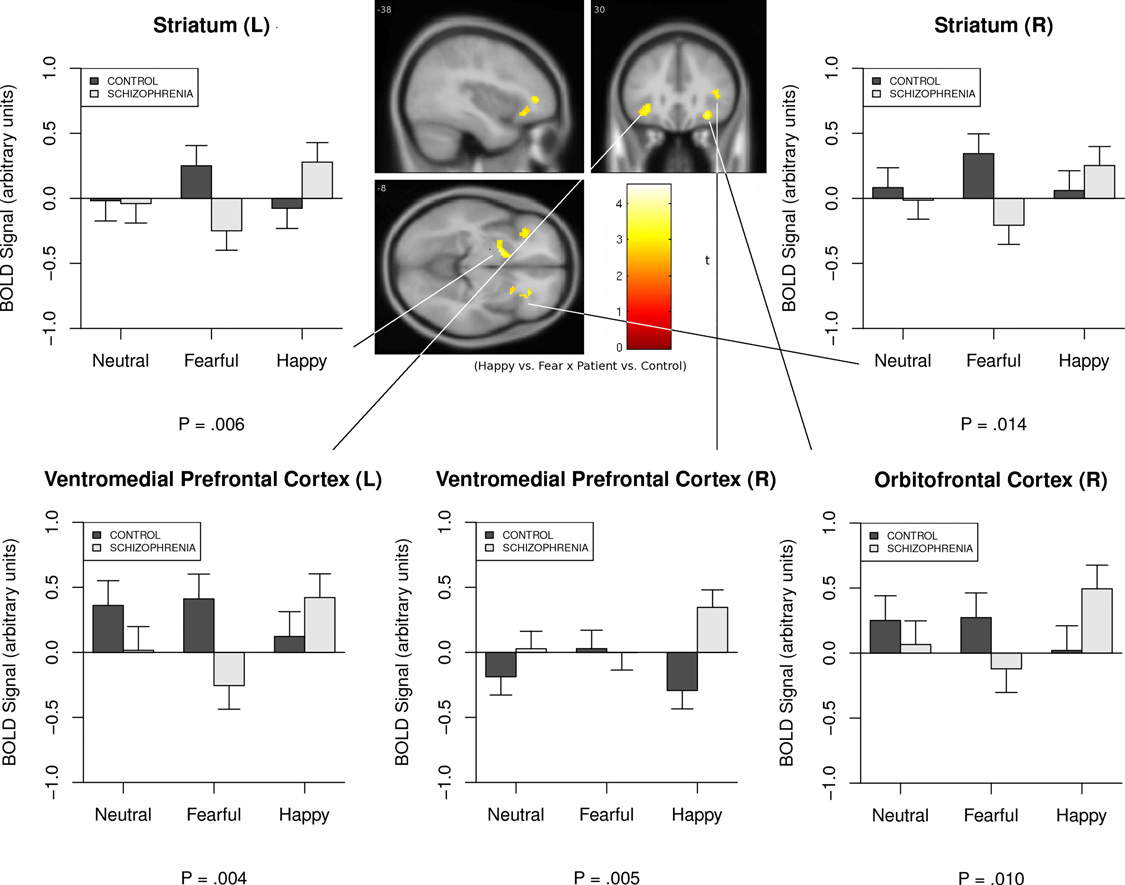
Fronto-Limbic Differences in Brain Activity Between Individuals with Schizophrenia and Healthy Volunteers During Voluntary Attentional Control of Positive Versus Negative Emotions.

Given the diametric pattern of responses to fearful and happy faces in patients with schizophrenia compared to healthy volunteers, the largest pattern of between-group differences emerged when directly contrasting BOLD signal response to these two emotional conditions. As can be seen in Fig 1, a clear disordinal interaction existed between fearful and happy conditions and participant group, such that patients with schizophrenia demonstrated reduced limbic activity in the bilateral striatum during the fearful face condition and increased striatal activity in the happy face condition. Conversely, the opposite pattern was observed in healthy volunteers, with increased striatal response during fearful trials and little to no differential striatal response during happy trials. Individuals with schizophrenia also displayed significant prefrontal hyperactivity in the bilateral ventromedial prefrontal cortex and right orbitofrontal cortex during happy versus fearful trials compared to controls. For the left ventromedial prefrontal cortex, this effect was at least partially driven by an inhibited prefrontal response to fearful trials. Taken together, these findings suggest significant fronto-limbic disengagement during the effortful processing of fearful emotions when completing a working memory task in schizophrenia, and an excessive striatal and prefrontal cortical response to the voluntary attentional control of positive emotional stimuli in the disorder.

### Behavioral Correlates of Fronto-Limbic Brain Function during Voluntary Attentional Control

**Fig 2:**
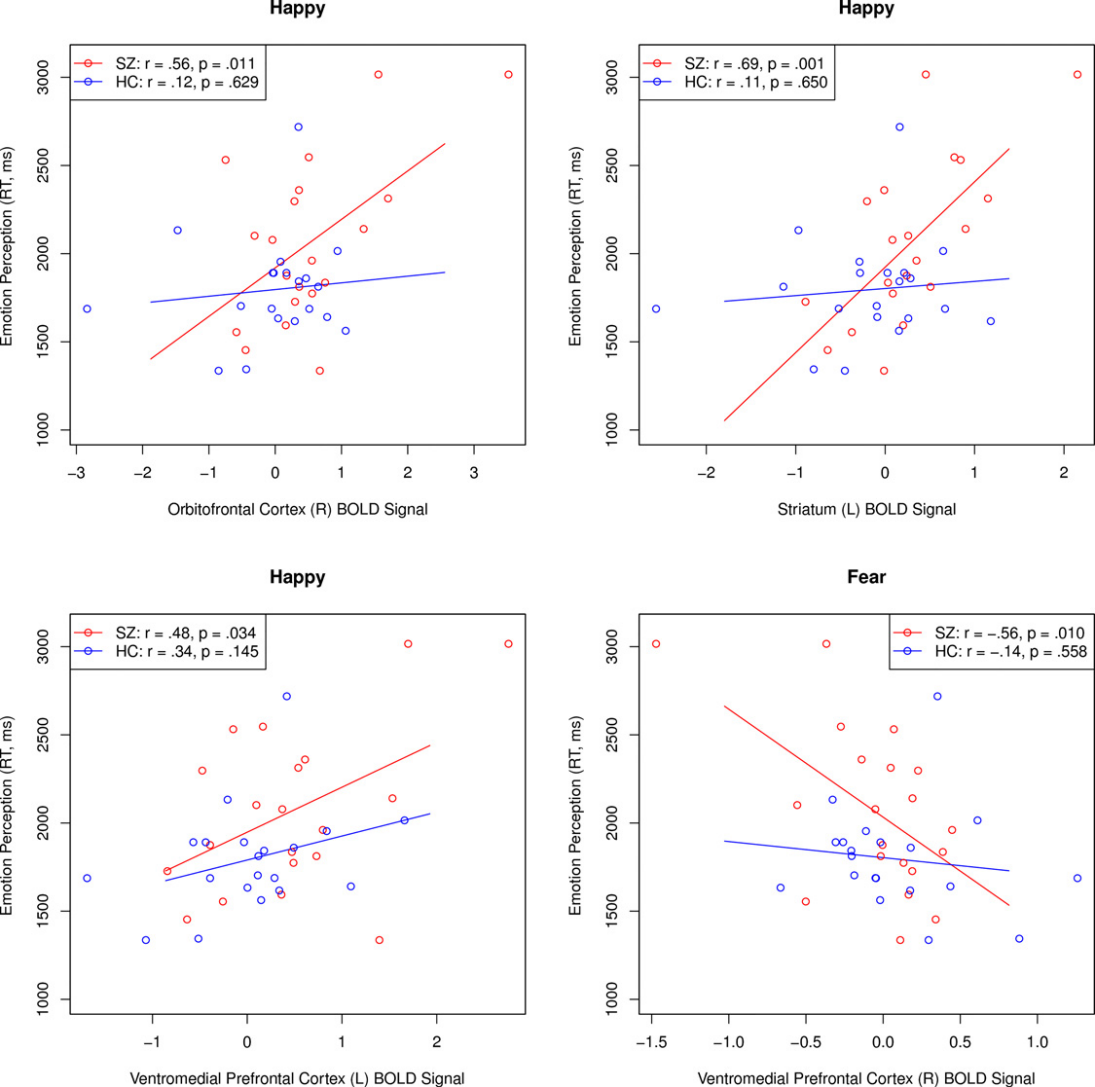
Associations Between Fronto-Limbic Brain Activity and Emotion Perception Reaction Time in People with Schizophrenia and Healthy Volunteers. HC = Healthy Control; SZ = Schizophrenia.

Having found that patients with schizophrenia displayed significant fronto-limbic dysregulation in response to fearful and happy face conditions, a series of within-group correlation analyses were conducted to examine the association between BOLD signal response and measures of emotion processing. With regard to task performance, increased left ventromedial prefrontal activity during happy face trials was associated with reduced accuracy on the emotional faces n-back task within the schizophrenia (*r* = -.55, *p* = .012), but not control group (*r* = .03, *p* = .747). Schizophrenia patients performed significantly worse than controls on the MSCEIT (*p* = .003) and were slower to respond (*p* = .032), but were not significantly less accurate on the Penn Emotion Recognition Test (*p* = .095). Although emotion perception accuracy was not significantly related to fronto-limbic brain activation in either patients or healthy volunteers, a consistent pattern of relationships was observed with regard to response time to recognize facial emotion expressions in patients (see Fig 2). Increased striatal, orbitofrontal, and ventromedial prefrontal cortical responses to happy faces were significantly associated with greater response latency on the Penn Emotion Recognition Test among individuals with schizophrenia, but not controls. Further, reduced right ventromedial prefrontal cortex activity in response to fearful faces was also related to slower response time in identifying facial expressions of emotion in those with the disorder, but not healthy individuals. When examining the MSCEIT, only reduced left striatal activity during happy face conditions was associated with increased emotional intelligence in healthy controls (*r* = -.54, *p* = .014), but not patients (*r* = .32, *p* = .164). Finally, no significant associations were observed between antipsychotic dosage and BOLD signal response in the striatum, ventromedial prefrontal, or right orbitofrontal cortex (mean |*r*| = .08, range of *r* = -.29 to .37).

## Discussion

Emotion dysregulation is a core characteristic of schizophrenia that is an important therapeutic target for improving recovery outcomes [1, 5]. This study examined abnormalities in the neural correlates associated with the voluntary attentional control subprocess of effortful emotion regulation in outpatients with schizophrenia and healthy volunteers using a well-established functional neuroimaging paradigm frequently applied in affective disorders [19]. Contrary to our hypothesis, patients with schizophrenia did not demonstrate fronto-limbic hyperfunction during the voluntary attentional control of negative emotions, but rather showed a *decrease* in BOLD signal activity in the bilateral striatum and left ventromedial prefrontal cortex in the presence of fearful faces. In contrast, when presented with happy face distractors, patients demonstrated hyperfunction in the striatum, ventromedial prefrontal, and right orbitofrontal cortex, whereas the opposite pattern of results was observed in healthy individuals, who tended to increase fronto-limbic activity in response to fearful faces and decrease such activity when presented with positive emotional distractors. Further, increased fronto-limbic activity during positive emotion conditions was related to greater latency of response during emotion perception in patients with schizophrenia, but not controls. Such findings may indicate significant fronto-limbic dysfunction during the voluntary attentional control subprocess of effortful regulation emotion in schizophrenia, with considerable striatal and prefrontal inefficiency in regulating positive emotions in the disorder.

The absence of prefrontal hyperactivity during the voluntary attentional control of negative emotions in this study is different than several recent investigations of the neural correlates of emotion regulation in schizophrenia [13]. Some studies that have observed prefrontal hyperfunction during the effortful regulation of negative stimuli have had an explicit focus on those stimuli for task completion (e.g., remember when a negative face was presented two trials previous), whereas emotional stimuli in this study were purely distracting, which may explain differences in the valance-specific findings. We tentatively hypothesize that when negative emotional stimuli are task-relevant, individuals with schizophrenia become fixated on those emotions and thus demonstrate an inefficient coordination of prefrontal resources, whereas when negative stimuli are not task-relevant, patients tend to quickly disengage from (and perhaps avoid orienting to) those stimuli facilitating task completion. Behavioral data from this study, while not significant, supports this hypothesis with patients completing negative trials the fastest (*M* = 645.50 ms) and positive trials the slowest (*M* = 698.02 ms), which was not the case for controls. Task-irrelevant positive stimuli appeared to be much more difficult for patients to regulate, and required significantly greater fronto-limbic resources, similar to what others have observed in affective disorders [34]. This pattern of results indicates a need for future studies to attend to challenges surrounding the effortful regulation of both positive and negative emotions in schizophrenia.

Despite the implications of this study for understanding the neural basis of effortful emotion regulation deficits in schizophrenia, these findings need to be understood within the context of several limitations. First, the sample size was modest, which may have precluded the detection of smaller effects in other brain regions (e.g., amygdala). Second, while participant groups were well-matched with regard to age and gender, they were not matched on race or IQ, which may have contributed to observed differences between schizophrenia patients and healthy volunteers. Between-group second-level fMRI models included these characteristics as confounding covariates to address this issue. Third, although antipsychotic dose was not related to BOLD signal response in any of the brain regions showing significant group differences, the prolonged and varied exposure to psychotropic medication among participants with schizophrenia represents an important potential source of influence on these findings. Future studies could profitably examine the degree to which significant fronto-limbic abnormalities persist among first-episode patients who have yet to receive long-term exposure to antipsychotic treatments. Fourth, a behavioral assessment of the perceived impact of emotional stimuli was not conducted, although numerous previous studies have shown consistent neural abnormalities to similar emotional faces in schizophrenia [35]. Further, BOLD signal responses to emotional distractors could reflect not only the voluntary control of attention during effortful emotion regulation, but also the general processing of emotional stimuli. It will be important for future studies to advance experimental paradigms that can disaggregate such processes. Finally, the sample was limited by diagnostic heterogeneity, with 45% of patients diagnosed with schizophrenia and the remaining with schizoaffective disorder. Caution should be used when interpreting the diagnostic specificity of these findings, as it is unclear whether the results of this research are specific to patients with schizophrenia, schizophrenia-spectrum disorders, or psychiatric disorders more generally. Several findings suggest that the abnormalities exhibited during emotion regulation in this sample of patients with schizophrenia and schizoaffective disorder may overlap substantially with those with bipolar and major depressive disorders [9], and it will be important to take a trans-diagnostic approach in future investigations, as neural deficits in the effortful regulation of emotion may represent a core deficit and therapeutic target across a variety of psychiatric disorders.

In summary, this study indicates that patients with schizophrenia may have significant fronto-limbic abnormalities when completing a task that requires the voluntary attentional control of emotional stimuli, with significant striatal and prefrontal disengagement during the regulation of negative emotions and significant hyperactivity in these same regions when processing positive emotional information. Despite limitations in diagnostic specificity, case-control matching, and sample size, these findings highlight the role of the striatum, ventromedial prefrontal, and orbitofrontal cortices in contributing to voluntary attentional control deficits in effortful emotion regulation in schizophrenia and suggest the importance of attending to and treating deficits in both effortful positive and negative emotion regulation in the disorder.

## References

[1] Kring AM, Elis O. Emotion deficits in people with schizophrenia. Annu Rev Clin Psychol. 2013;9:409–433. 10.1146/annurev-clinpsy-050212-185538 23245340

[2] Marwaha S, Broome MR, Bebbington PE, Kuipers E, Freeman D. Mood instability and psychosis: Analyses of British national survey data. Schizophr Bull. 2014;40:269–277. 10.1093/schbul/sbt149 24162517 PMC3932088

[3] Zubin J, & Spring B. Vulnerability: A new view of schizophrenia. J Abnorm Psychol. 1977;86:103–126. 858828 10.1037//0021-843x.86.2.103

[4] Berenbaum H, Oltmanns TF. Emotional experience and expression in schizophrenia and depression. J Abnorm Psychol. 1992;101:37–44. 1537971 10.1037//0021-843x.101.1.37 PMC4370316

[5] O’Driscoll C, Laing J, Mason O. Cognitive emotion regulation strategies, alexithymia and dissociation in schizophrenia, a review and meta-analysis. Clin Psychol Rev. 2014;34:482–495. 10.1016/j.cpr.2014.07.002 25105273

[6] Gumora G, Arsenio WF. Emotionality, emotion regulation, and school performance in middle school children. Journal of School Psychology. 2002;40:395–413.

[7] Gross JJ, John OP. I ndividual differences in two emotion regulation processes: Implications for affect, relationships, and well-being. J Pers Soc Psychol. 2003;85:348–362. 12916575 10.1037/0022-3514.85.2.348

[8] Gross JJ. The emerging field of emotion regulation: An integrative review. Rev Gen Psychol. 1998;2:271–299.

[9] Phillips ML, Ladouceur CD, Drevets WC. A neural model of voluntary and automatic emotion regulation: Implications for understanding the pathophysiology and neurodevelopment of bipolar disorder. Mol Psychiatry. 2008;13:833–857. 10.1038/mp.2008.65 PMC2745893 18574483

[10] Gyurak A, Gross JJ, Etkin A. Explicit and implicit emotion regulation: A dual-process framework. Cognition and Emotion. 2011;25:400–412. 10.1080/02699931.2010.544160 21432682 PMC3280343

[11] Heinrichs RW, Zakzanis KK. Neurocognitive deficit in schizophrenia: A quantitative review of the evidence. Neuropsychology. 1998;12:426–445. 9673998 10.1037//0894-4105.12.3.426

[12] Webb TL, Miles E, Sheeran P. Dealing with feeling: A meta-analysis of the effectiveness of strategies derived from the process model of emotion regulation. Psychol Bull. 2012;138:775–808. 10.1037/a0027600 22582737

[13] Becerril K, Barch D. Influence of emotional processing on working memory in schizophrenia. Schizophr Bull. 2011;37:1027–1038. 10.1093/schbul/sbq009 20176860 PMC3160211

[14] Anticevic A, Repovs G, Corlett PR, Barch DM. Negative and nonemotional interference with visual working memory in schizophrenia. Biol Psychiatry. 2011;70:1159–1168. 10.1016/j.biopsych.2011.07.010 21861986

[15] Anticevic A, Repovs G, Barch DM. Emotion effects on attention, amygdala activation, and functional connectivity in schizophrenia. Schizophr Bull. 2012;38:967–980. 10.1093/schbul/sbq168 21415225 PMC3446234

[16] Anticevic A, Repovs G, Krystal JH, Barch DM. A broken filter: prefrontal functional connectivity abnormalities in schizophrenia during working memory interference. Schizophr Res. 2012;141:8–14. 10.1016/j.schres.2012.07.007 22863548 PMC3879404

[17] van der Meer L, Swart M, van der Velde J, Pijnenborg G, Wiersma D, Bruggeman R, et al Neural correlates of emotion regulation in patients with schizophrenia and non-affected siblings. PLoS One. 2014;9:e99667 10.1371/journal.pone.0099667 24941136 PMC4062465

[18] Kohler CG, Walker JB, Martin EA, Healey KM, Moberg PJ. Facial emotion perception in schizophrenia: a meta-analytic review. Schizophr Bull. 2010;36:1009–1019. 10.1093/schbul/sbn192 19329561 PMC2930336

[19] Ladouceur CD, Dahl RE, Williamson DE, Birmaher B, Ryan ND, Casey BJ. Altered emotional processing in pediatric anxiety, depression, and comorbid anxiety-depression. J Abnorm Child Psychol. 2005;33:165–177. 15839495 10.1007/s10802-005-1825-z

[20] Ladouceur CD, Silk JS, Dahl RE, Ostapenko L, Kronhaus DM, Phillips ML. Fearful faces influence attentional control processes in anxious youth and adults. Emotion. 2009;9:855–864. 10.1037/a0017747 20001128

[21] First MB, Spitzer RL, Gibbon M, Williams JBW. Structured Clinical Interview For DSM-IV-TR Axis I Disorders, Research Version, Patient Edition New York: Biometrics Research, New York State Psychiatric Institute; 2002.

[22] Tottenham N, Tanaka JW, Leon AC, McCarry T, Nurse M, Hare TA, et al The NimStim set of facial expressions: Judgments from untrained research participants. Psychiatry Res. 2009;168:242–249. 10.1016/j.psychres.2008.05.006 19564050 PMC3474329

[23] Kohler CG, Turner TH, Bilker WB, Brensinger CM, Siegel SJ, Kanes SJ, et al Facial Emotion Recognition in Schizophrenia: Intensity Effects and Error Pattern. Am J Psychiatry. 2003;160:1768–1774. 14514489 10.1176/appi.ajp.160.10.1768

[24] Mayer JD, Salovey P, Caruso DR, Sitarenios G. Measuring emotional intelligence with the MSCEIT V2.0. Emotion. 2003;3:97–105. 12899321 10.1037/1528-3542.3.1.97

[25] Salovey P, Mayer JD. Emotional Intelligence. Imagin Cogn Pers. 1990;9:185–221.

[26] Carter CS, Barch DM, Gur R, Gur R, Pinkham A, Ochsner K. CNTRICS Final Task Selection: Social Cognitive and Affective Neuroscience-Based Measures. Schizophr Bull. 2009;35:153–162. 10.1093/schbul/sbn157 19011231 PMC2643972

[27] Eack SM, Greeno CG, Pogue-Geile MF, Newhill CE, Hogarty GE, Keshavan MS. Assessing social-cognitive deficits in schizophrenia with the Mayer-Salovey-Caruso Emotional Intelligence Test. Schizophr Bull. 2010;36:370–380. 10.1093/schbul/sbn091 18648021 PMC2833113

[28] Nuechterlein KH, Green MF, Kern RS, Baade LE, Barch DM, Cohen JD, et al The MATRICS Consensus Cognitive Battery, Part 1: Test Selection, Reliability, and Validity. Am J Psychiatry. 2008;165:203–213. 10.1176/appi.ajp.2007.07010042 18172019

[29] Ammons RB, Ammons CH. The Quick Test (QT): provisional manual. Psychol Rep. 1962;11:111–161.

[30] Whitfield-Gabrieli S. Artifact Detection Tools. Boston, MA: Massachusetts Institute of Technology; 2011.

[31] Maldjian JA, Laurienti PJ, Kraft RA, Burdette JH. An automated method for neuroanatomic and cytoarchitectonic atlas-based interrogation of fMRI data sets. Neuroimage. 2003;19:1233–1239. 12880848 10.1016/s1053-8119(03)00169-1

[32] Tzourio-Mazoyer N, Landeau B, Papthanassiou D, Crivello F, Etard O, Delcroix N, et al Automated anatomical labeling of activations in SPM using a macroscopic anatomical parcellation of the MNI MRI single-subject brain. Neuroimage. 2002;15:273–289. 11771995 10.1006/nimg.2001.0978

[33] Ward DB. Simultaneous inference for fMRI data. Milwaukee, WI: Author; 2000.

[34] Bertocci MA, Bebko GM, Mullin BC, Langenecker SA, Ladouceur CD, Almeida JRC, et al Abnormal anterior cingulate cortical activity during emotional n-back task performance distinguishes bipolar from unipolar depressed females. Psychol Med. 2012;42:1417–1428. 10.1017/S003329171100242X 22099606 PMC3601380

[35] Taylor SF, Kang J, Brege IS, Tso IF, Hosanagar A, Johnson TD. Meta-analysis of functional neuroimaging studies of emotion perception and experience in schizophrenia. Biol Psychiatry. 2012;71:136–145. 10.1016/j.biopsych.2011.09.007 21993193 PMC3237865

[36] Overall JE, Gorham DR. The Brief Psychiatric Rating Scale. Psychol Rep. 1962;10:799–812.

[37] Endicott J, Spitzer RL, Fleiss JL, Cohen J. The Global Assessment Scale: A procedure for measuring overall severity of psychiatric disturbance. Arch Gen Psychiatry. 1976;33:766–771. 938196 10.1001/archpsyc.1976.01770060086012

